# Rhythmic abilities and musical training in Parkinson’s disease: do they help?

**DOI:** 10.1038/s41531-018-0043-7

**Published:** 2018-03-23

**Authors:** V. Cochen De Cock, D. G. Dotov, P. Ihalainen, V. Bégel, F. Galtier, C. Lebrun, M. C. Picot, V. Driss, N. Landragin, C. Geny, B. Bardy, S. Dalla Bella

**Affiliations:** 1 Department of Neurology, Beau Soleil Clinic, Montpellier, France; 2 Clinical Investigation Centre (CIC), 1411, University hospital of Montpellier and Inserm, Montpellier, France; 3 EuroMov, Montpellier University, Montpellier, France; 4 Clinique du Millénaire, Montpellier, France; 5 Department of Neurology, University Hospital of Montpellier, Montpellier, France; 6 Department of Psychology, University of Montreal, Montreal, Canada; 7 International Laboratory for Brain, Music, and Sound Research (BRAMS), Montreal, Canada; 8 Department of Cognitive Psychology, WSFiZ, Warsaw, Poland

## Abstract

Rhythmic auditory cues can immediately improve gait in Parkinson’s disease. However, this effect varies considerably across patients. The factors associated with this individual variability are not known to date. Patients’ rhythmic abilities and musicality (e.g., perceptual and singing abilities, emotional response to music, and musical training) may foster a positive response to rhythmic cues. To examine this hypothesis, we measured gait at baseline and with rhythmic cues in 39 non-demented patients with Parkinson’s disease and 39 matched healthy controls. Cognition, rhythmic abilities and general musicality were assessed. A response to cueing was qualified as positive when the stimulation led to a clinically meaningful increase in gait speed. We observed that patients with positive response to cueing (*n* = 17) were more musically trained, aligned more often their steps to the rhythmic cues while walking, and showed better music perception as well as poorer cognitive flexibility than patients with non-positive response (*n* = 22). Gait performance with rhythmic cues worsened in six patients. We concluded that rhythmic and musical skills, which can be modulated by musical training, may increase beneficial effects of rhythmic auditory cueing in Parkinson’s disease. Screening patients in terms of musical/rhythmic abilities and musical training may allow teasing apart patients who are likely to benefit from cueing from those who may worsen their performance due to the stimulation.

## Musicality in Parkinson’s disease: Trained to follow the beat

Listening to rhythmic auditory cues improves the ability to walk in patients with Parkinson’s disease (PD). Previous studies have shown that music can help patients with neurological disorders synchronize their movements to a beat. An international study led by Valérie Cochen De Cock at Clinique Beau Soleil in Montpellier (France) and Simone Dalla Bella at the International Laboratory For Brain, Music and Sound Research (BRAMS) in Montreal (Canada), measured gait speed in 39 patients with PD without dementia in response to rhythmic stimulation. Twenty-two patients increased their gait speed by spontaneously synchronizing their steps to the beat. The remaining 17 patients showed no effect or significant worsening of gait performance (e.g., smaller step length). The patients who benefited the most from rhythmic cues exhibited better rhythmic skills and were more musical than the others. Assessing musical abilities may serve to identify patients who are likely to benefit from this music-based intervention and may foster individualization of the treatment.

## Introduction

Music is a universal trait of humankind. The majority can move to the beat, react emotionally to music, recognize well-known tunes, and sing proficiently. These skills, which can be improved via dedicated training, are generally referred to as “musicality”,^1^ and vary considerably among individuals. Remarkably, individual differences in musicality may play a critical role in understanding the variability of the response to music-based interventions in neurological rehabilitation.^2^ In particular, rhythmic skills and the ability to move to the beat of music may predict the well-known response to rhythmic auditory cueing (RAC) on gait of patients with Parkinson’s Disease (PD).^3–5^ In PD, the dysfunctional basal-ganglia-cortical circuitry is associated with timing distortions in the perception and production of rhythmic events.^6–9^ Providing an external rhythmic cue is likely to compensate for the impaired internal generation of rhythm, as suggested recently.^4^ The magnitude of this effect and whether RAC improves or deteriorates motor performance may depend on individual differences in rhythmic skills.

In patients with PD, the immediate beneficial effect of RAC on gait (increased speed, stride length and reduction of freezing episodes) has largely been demonstrated.^10–15^ However, these effects have only been described at the group level. Even though the effect of stimulation can vary significantly form one study to the other (e.g., with average effect sizes for stride length between 0 and 0.5),^16^ individual variability of this response and its determinants have not been examined so far, nor the possibility of deleterious effects of cueing in some patients. Finally, oftentimes music is used by the general population to improve motivation and performance in motor activities, such as in sport.^17^ For example, most people run and walk while using music-based applications implemented in mobile devices. Similar music-based applications are already proposed to patients^18^ but the risks associated with the potential deleterious effects of music delivered by these technologies in patients with PD have never been addressed.

To date there are no guidelines for using RAC as an individualized clinical tool. The ability to track the beat of rhythmic cues may allow predicting a patient’s response to cueing, as suggested for healthy young adults.^19^ Moreover, other aspects of musicality such as perceptual skills, emotional response to music, and musical training, as well as clinical and cognitive functioning may also modulate the beneficial effect of cueing. To this end, we examined patients’ individual gait response to various rhythmic stimuli, tested their motor and non-motor rhythmic performance, and assessed their general musicality. The differences between patients with positive response (PR) and non-positive response (NPR) to cueing were examined. The ultimate goal was to provide guidelines to identify patients who will most likely benefit from RAC, while excluding those patients who are at risk of seeing their performance worsened by cueing.

## Results

### Clinical and neuropsychological evaluations

Clinical and neuropsychological evaluations of patients and controls are presented in Table 1. Patients were comparable to controls in terms of general cognition (Montreal Cognitive Assessment score). However, patients exhibited more depressive symptoms, and more apathy than controls. Moreover, they were more concerned than controls by the fact that they might fall.

**Table 1:** Clinical characteristics, cognition and psychopathological evaluation of patients with Parkinson’s disease with positive and no positive response to cueing and controls

	Controls	Patients with PD			Patients vs. controls	Patients with PD, PR vs. NPR
		All	Positive response (PR)	Non-positive response (NPR)	*p*	*p*
Participants (*n*)	39	39	22	17		
Age	62 ± 10	62 ± 10	65 ± 11	60 ± 8	1	0.25
Gender (number of males)	24	24	11	13	1	0.6
Disease duration (years)	–	8 ± 5	8 ± 4	9 ± 6	–	
Age at onset	–	54 ± 10	56 ± 11	51 ± 8	–	0.16
LEDD	0	909 ± 496	772 ± 367	948 ± 604	–	0.3
Hoehn and Yahr	0	2.0 ± 0.5	2.0 ± 0.6	1.9 ± 0.4	<0.001	0.3
MDS-UPDRS-III	2.3 ± 2.9	24.3 ± 13.2	26.1 ± 15.9	21.9 ± 8.4	<0.001	0.3
Falls Self Efficacy Scale Score	7.4 ± 1.2	11.1 ± 3.8	11.0 ± 3.5	11.3 ± 4.2	<0.001	0.8
Axial signs	0.4 ± 0.5	3.6 ± 2.2	3.9 ± 2.5	3.24 ± 1.8	<0.001	0.7
MDS-UPDRS-I	3.23 ± 2.4	11.5 ± 6.4	11.1 ± 6.3	11.8 ± 6.6	<0.001	0.7
MDS-UPDRS-II	0.76 ± 3.2	11.6 ± 5.5	11.3 ± 5.3	12.0 ± 5.9	<0.001	0.7
MDS-UPDRS-IV	0.0 ± 0.0	3.1 ± 2.9	2.1 ± 2.7	4.55 ± 2.8	<0.001	0.5
MOCA	27.5 ± 1.9	27.2 ± 2.3	26.8 ± 3.2	27.7 ± 2.1	0.6	0.1
Apathy (lars)	–11.4 ± 2.4	−9.8 ± 3.5	–10.1 ± 2.8	−9.5 ± 4.2	0.02	0.3
Depression (BDI)	5.7 ± 6.4	13.7 ± 9.2	13.0 ± 9.5	14.6 ± 9.1	<0.01	0.3
Working memory (WAIS digit span)	11.2 ± 2.5	10.2 ± 2.9	9.9 ± 3.1	10.5 ± 2.6	0.1	0.2
Cognitive flexibility						
Trail making test A	37.3 ± 19.3	50.1 ± 39.5	59.2 ± 50.6	38.7 ± 12.5	0.07	0.08
Trail making test B	89.8 ± 30.0	129.8 ± 89.1	136.0 ± 97.4	122.1 ± 79.9	0.01	0.63
B/A ratio	2.6 ± 0.7	2.8 ± 1.1	2.6 ± 0.8	3.1 ± 1.4	0.3	0.1
Wisconsin						
Number of catergories	5.7 ± 0.6	4.8 ± 1.4	4.6 ± 1.6	5.1 ± 1.1	0.002	0.2
Number of errors	6.4 ± 4.2	10.5 ± 7.0	12.0 ± 7.6	8.4 ± 5.6	0.003	0.1
Number of perseverations	1.6 ± 1.7	3.0 ± 3.3	3.8 ± 3.8	1.9 ± 2.0	0.03	0.05
Inhibition (stroop)						
Naming raw time	60.9 ± 11.2	71.5 ± 18.7	74.9 ± 21.3	66.9 ± 13.7	0.003	0.2
Reading raw time	42.9 ± 6.6	49.8 ± 11.9	50.6 ± 13.4	48.8 ± 9.8	0.002	0.6
Interference raw time	115.5 ± 32.7	144.5 ± 83.2	159.4 ± 99.7	125.1 ± 52.0	0.05	0.2
Naming score	42.3 ± 16.9	45.0 ± 26.6	49.6 ± 27.5	39.1 ± 25.0	0.6	0.2
Interefence score	89.5 ± 34.1	96.4 ± 59.5	103.7 ± 58.5	86.8 ± 61.3	0.5	0.4

### Effects of RAC on gait

At baseline, patients exhibited lower gait velocity and bilateral coordination, shorter stride length, and higher gait variability than controls (Table 2). The two groups were comparable in terms of cadence. Patients and controls increased their cadence and their velocity in trials with cueing.

**Table 2:** Cueing effect on gait parameters in patients with Parkinson’s disease and controls

	Patients with PD (*n* = 39)		Controls (*n* = 39)		Patients vs. controls		Cueing vs. baseline	
	Baseline	Cueing	Baseline	Cueing	*F*(df)	*p*	*F*(df)	*p*
Cadence (steps/min)	107.04 ± 12.94	113.09 ± 11.25	106.46 ± 8.31	109.25 ± 8.63	1.17 (1,76)	0.3	13.63 (1,76)	<0.001
Velocity (m/s)	1.13 ± 0.15^a^	1.21 ± 0.15	1.26 ± 0.11	1.32 ± 0.11	22.90 (1,76)	<0.001	18.04 (1,76)	<0.001
Stride length (m)	1.27 ± 0.15^a^	1.30 ± 0.16	1.43 ± 0.10	1.45 ± 0.10	39.39(1,76)	<0.001	2.57 (1,76)	0.2
Gait Variability (CV stride)	0.025 ± 0.011^a^	0.026 ± 0.007	0.020 ± 0.005	0.021 ± 0.006	14.16(1,76)	<0.001	1.63 (1,76)	0.1
Coordination index (PCI, %)	4.72 ± 2.06^a^	4.97 ± 2.05	3.75 ± 1.28	3.81 ± 1.16	9.89(1,76)	0.02	0.77(1,76)	0.4

*CV stride* coefficient of variation of the inter-stride interval (standard deviation of the inter-stride intervals divided by the mean inter-stride interval), *PCI* phase coordination index, *df* degrees of freedom

^a^For a difference between patients and controls at pre-test

**Fig. 1:**
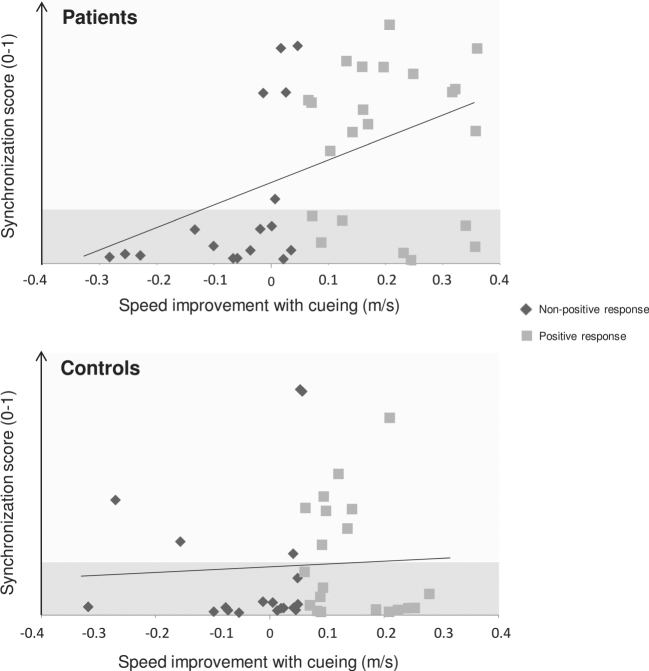
Individual responses to rhythmic cueing expressed as the difference in gait speed between cueing and the baseline, in patients with Parkinson's disease and controls. Patients who aligned their steps to the beat also increased their speed; this is not the case of controls

In spite of the aforementioned group effects of RAC, there were important inter-individual differences (Fig. 1). Participants were divided into two categories based on their response to RAC relative to the baseline.^4^ An improvement in gait speed larger than the smallest clinically significant difference in PD (0.06 m/s) characterized participants with a PR to cueing.^20^ Participants with a smaller or a negative difference were considered as participants with a NPR. Finally, a reduction in gait speed by more than the smallest clinically significant difference in PD (0.06 m/s)^20^ characterized participants with a negative response (NR) to cueing.

Twenty-two patients and 20 controls had a PR to cueing, while 17 patients and 19 controls showed a NPR. In particular, six patients and six controls showed a NR with a significant worsening of gait performance (−0.18 ± 0.09 m/s and −0.17 ± 0.11 m/s, respectively). Patients spontaneously synchronized their steps to the beat more often than controls did (synchronization score: 0.40 ± 0.34 vs. 0.20 ± 0.26, respectively; *t*(73.7) = 3.01, *p* < 0.01). Better step synchronization to the beat was associated to greater improvement with cueing in patients (*R*^2^ = 0.16, *F*(1, 37) = 7.1, *p* = 0.01) but not in controls (*R*^2^ = 0.002, *F* < 1, *p* = 0.78).

### Individual differences (PR vs. NPR to cueing)

#### Clinical and neuropsychological tests

Patients with PR and NPR did not differ in terms of age, disease duration, age at disease onset, and levodopa-equivalent daily dose. No difference between the two sub-groups was found for the severity of motor symptoms (Table 1). Control participants with PR and NPR to cueing did not differ on any measure (*p*s > 0.14).

In the neuropsychological evaluation (see Table 1), patients with PR to cueing were the most impaired in terms of cognitive flexibility, tested with the Wisconsin Card Sorting Task. They identified the same number of categories, did the same number of errors but showed significantly more perseverations (*p* = 0.05) as compared with patients with NPR. In spite of this difference, patients with PR and NPR to cueing did not significantly differ in global cognition, working memory and inhibition. They also did not differ on the psychopathological assessment of depression and apathy.

#### Gait parameters

**Fig. 2:**
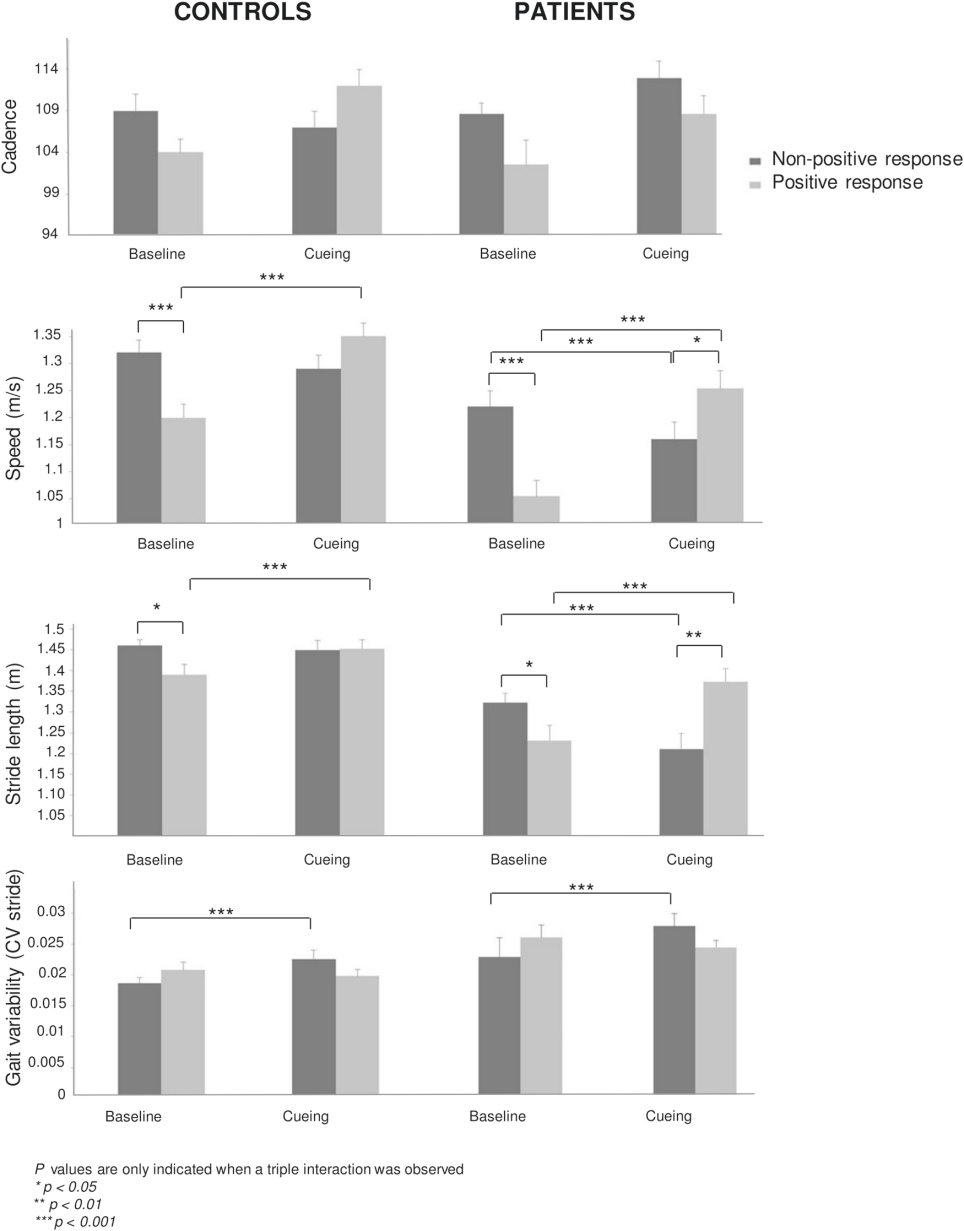
Spatio-temporal gait parameters in patients with Parkinson's diseaseand controls at baseline and with cueing. Participants are divided into two categories depending on their response to cueing (positive vs. non-positive). In patients with positive response speed and stride length improved while in patients with non-positive response both worsened. Error bars indicate standard deviation

Patients and controls both engaged in the task as shown by the increase of cadence in both groups. This effect was however more apparent in participants with a PR to cueing than in those with a NPR (*F*(1,74) = 6.01, *p* < 0.05; Fig. 2).

As expected, patients and controls with PR improved significantly their gait speed with cueing. The improvement in patients with PR was such (+19 % relative to the baseline), that they reached the speed of controls at baseline (1.26 m/s, for the overall group, *t* < 1). Gait speed at baseline in patients and controls with PR was lower than in participants with NPR to cueing. Participants with PR to cueing had room for improvement, thus avoiding a ceiling effect. Interestingly, patients with NPR, in spite of their less impaired performance without cues, did not maintain their gait speed with cues, but rather showed worsening of their performance (lower gait speed by −5%).

Similar effects were observed for stride length. At baseline, patients and controls with PR showed shorter strides than participants with NPR to cueing. Patients and controls with PR significantly increased their stride length with cueing (by 14 cm and 6 cm, respectively), while only patients with NPR exhibited a deleterious effect of cueing on stride length, significantly smaller when walking with cues (by 11 cm).

Finally, in both patients and controls with NPR, cueing had a deleterious effect on stride length variability, increased relatively to the baseline (*F*(1,74) = 8.10 *p* < 0.01).

#### Rhythm perception

**Fig. 3:**
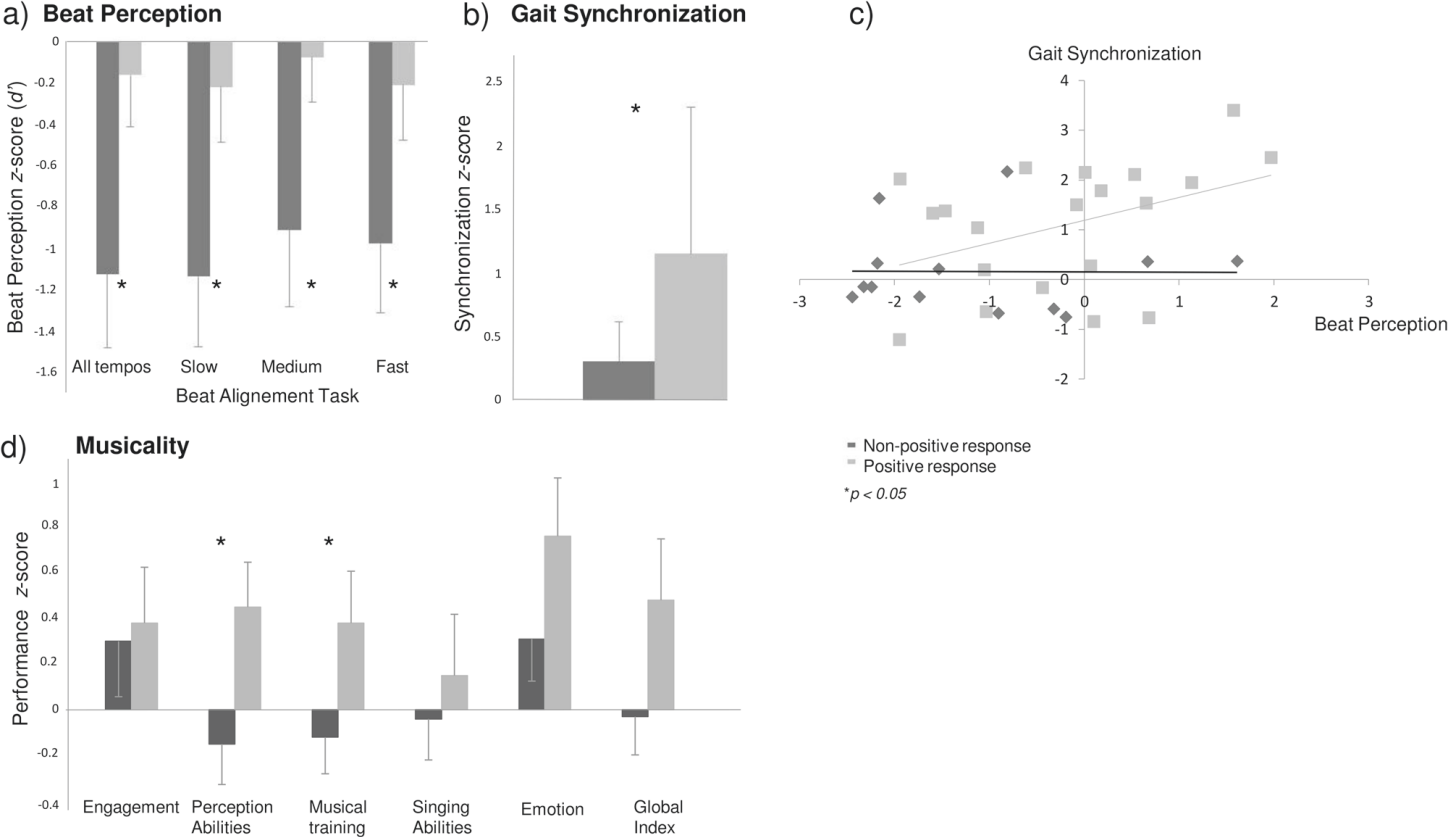
**a** Beat perception, **b** Gait synchronization to auditory cues, **c** Correlation between beat perception and gait synchronization, and **d** Musicality in patients with PD with positive and non-positive response to cueing. In patients with positive response, beat perception is relatively spared, and the alignment of steps to the beat, perceptual abilities, and musical training are higher than in patients with non-positive response. Error bars indicate standard deviation

Beat perception in patients with PR to cueing was not altered since they did not differ from controls (mean *d’* for patients = 1.96 ± 1.28, *p* > 0.50). In contrast, patients with NPR to cueing revealed poor beat perception, in the overall performance and across the different tempos. Control participants with PR and NPR to cueing (Fig. 3a) did not differ on the Beat Alignment Test (mean *d*’ = 2.14 ± 0.99, *p*s > 0.45).

#### Rhythm production

No difference between control participants with PR and NPR was found in the unpaced tapping task (inter-tap interval: 731.26 ± 236.56 ms; motor variability: 0.04 ± 0.01, *p*s > 0.52). Patients showed slightly greater motor variability than controls (variability for patients: 0.06 ± 0.03, *t*(49.4) = 3.42, *p* < 0.01). Yet, patients with PR and NPR did not differ on these measures of rhythm production (*z*-scores for inter-tap intervals: −0.23 ± 1.50 vs. 0.35 ± 1.13; motor variability: −1.43 ± 2.36 vs. −1.55 ± 2.86; *p*s > 0.44).

#### Synchronization to the beat (in gait and tapping)

Patients with PR to cueing aligned their steps to the beat significantly better than the other patients (*t*(37) = 2.35, *p* = 0.01, Fig. 3b). Interestingly, better synchronization to the beat was positively associated to beat perception among patients with PR to cueing (*r* = 0.42, *p* < 0.05). Patients with better beat perception and better synchronization of their steps to the beat benefited most from rhythmic cues (Fig. 3c). Finally, patients who stepped to the beat were those who obtained the lowest scores in terms of cognitive flexibility (Wisconsin Card Sorting Task, n. of categories, *r* = −0.37, *p* < .05; n. of errors, *r* = 0.43, *p* < 0.01; n. of perseverations, *r* = 0.36, *p* < 0.05).

In the paced tapping task, control participants with PR and NPR to cueing did not differ (synchronization score, with the metronome: 0.95 ± 0.08; with music: 0.86 ± 0.20; *p*s > 0.80). In general patients showed slightly lower synchronization scores than controls, only with the metronome (0.93 ± 0.09 vs. 0.95 ± 0.08, *t*(71.3) = 1.73, *p* < 0.05). No differences were found with music (0.84 ± 0.20 vs. 0.86 ± 0.19, *p* = 0.21, respectively). Comparison of synchronization scores for patients with PR and NPR to cueing revealed no significant differences between the two sub-groups (mean *z*-scores, with the metronome:−0.57 ± 1.26 vs. −0.23 ± 0.83; with music:−0.11 ± 0.86 vs. −0.28 ± 1.12, respectively; *p*s > 0.29).

#### Musicality

Patients did not differ from controls overall in terms of musicality (Gold-MSI global index: 58.26 ± 15.60 vs. 55.24 ± 17.8, respectively, *p* = 0.4). Control participants with PR and NPR to cueing did not differ in any of the measures of the Gold-MSI (mean performance: 55.40 ± 17.6, *p* = 0.20).

Patients with PR to cueing (Fig. 3d) showed higher scores for perceptual abilities (*t*(36) = 2.28, *p* < 0.05), and musical training (*t*(34.1) = 1.80, *p* < 0.05) than patients with NPR; a trend towards significance was found for the Gold-MSI global index (*t*(32.5) = 1.61, *p* = 0.06). In contrast, no differences between the two sub-groups were found for the other measures (active engagement with music, singing abilities, and emotion). Thus, highly musical patients, namely in terms of self-assessed perceptual skills and musical training, were the ones who mostly benefited from cueing.

#### NR to cueing

The comparison between patients with a NR to cueing (*n* = 6) and the other patients (*n* = 33) revealed that patients with NR walked faster at baseline than the other patients (1.27 ± 0.1 vs. 1.1 ± 0.1, *p* < 0.01) and were the least impaired in terms of cognitive flexibility, tested with the Wisconsin Card Sorting Test. Patients with NR identified more categories (6.0 ± 0.0 vs. 4.6 ± 1.5, *p* = 0.04), made less errors (3.8 ± 3.1 vs. 11.5 ± 6.9, *p* = 0.01), and less perseverations (0.4 ± 0.5 vs. 3.4 ± 3.3, *p* = 0.01) as compared with the other patients. In addition, patients with NR had lower scores in the Gold-MSI for musical training and perceptual abilities (Gold-MSI *z*-score, respectively, for musical training: −0.5 ± 0.4 vs. 0.3 ± 0.9, *p* = 0.05; and perceptual abilities: −0.5 ± 0.6 vs. 0.3 ± 0.9, *p* = 0.04), and aligned their steps to the beat less precisely than the other patients (−0.27 ± 0.36 vs. 0.98 ± 1.2, *p* = 0.03). They did not significantly differ from the other patients for all the other measures of gait, cognition, and musicality (*ps* > 0.07). Interestingly, these results show that the characteristics of the patients with NR to cueing are the opposite of what observed in patients with PR.

#### Prediction of a PR or NPR to cueing

Patients’ subgroup (NPR vs. PR to cueing) was entered as a binary dependent variable (0/1) into a logistic regression model. We report here the model leading to the most satisfactory fit to the data after examining various models testing predictors which showed significant differences between the two sub-groups of patients. Gait velocity at baseline (without auditory stimulation), the overall performance in the Beat Alignment Test, and the number of perseverations obtained in the Wisconsin Card Sorting Test were the significant predictors. Note that gait synchronization and musicality (the performance obtained with the Gold-MSI) were also initially entered in the full model, but as these predictors did not significantly improve the model fit, they were removed from the final model. The model provides a highly significant fit as compared to a null model (null −2LL = 7.7, final −2LL = 21.2, *χ*^*2*^ = 26.9, *p* < .0001; Nagelkerke *R*^*2*^ = .77; AIC = 23.5). The model indicates that lower velocity at baseline (*B* = −28.1, *SE*(*B*) = 12.5, Wald test = −2.25, *p* < 0.05), better beat perception as revealed by the Beat Alignment Test (*B* = 1.3, *SE*(*B*) = 0.6, Wald test = 2.01, *p* < 0.05), and worse performance in the Wisconsin Card Sorting Test (*B* = 0.6, *SE*(*B*) = 0.3, Wald test = 1.91, *p* = 0.06) all increase the probability of a PR to cueing.

## Discussion

Patients with PD responded very differently to RAC. Some of them significantly increased their speed and did longer steps, while others in spite of relatively unimpaired gait without the stimulation, worsened dramatically their performance. In some cases RAC can thus hamper gait kinematics, a result which is at odds with the generally observed beneficial effects of the stimulation.^12–14,19^ We uncovered factors linked to PR or NPR pertaining to rhythmic abilities and other aspects of musicality (i.e., perception and musical training), baseline gait performance, and cognitive functioning.

Patients who positively responded to cueing could track the beat of an auditory stimulus, while patients with NPR struggled with beat perception. This ability was linked to patients’ ability to synchronize their steps to the beat of music while walking with cues. Notably, this finding is not merely the result of instructions, as participants were not explicitly told to synchronize their steps to the beat. Rather, it is likely that patients can positively respond to cueing because they can still capitalize on spared beat perception and synchronization mechanisms. PR to cueing was also associated with some aspects of patients’ musicality.^1^ Fine grained perceptual skills and musical training positively affected the response to cueing. Surprisingly, the patients who benefited most from the cues were also those with poorer cognitive flexibility.

In spite of the oft-reported timing and rhythmic deficits in PD,^6–8^ many patients could still synchronize to the beat, a fact which was associated to a PR to the cues. In contrast, patients who were unable to align their steps to the beat may have found themselves in a dual-task situation whereby rhythmic cues rather disturbed gait. The deleterious effect of dual tasks on gait is well known in PD.^21^

Significantly, controls and almost one third of the patients benefited from cueing even if they did not align their steps to the beat. Other factors such as emotional and motivational aspects may also contribute to improve gait.^2,22^ Music is typically a motivating stimulus, known for its ability to engage emotions and stimulate the reward system, while acting on the dopaminergic system.^23,24^ Walking with music may be a rewarding activity in itself. However, our results show no influence of the type of stimulus. Walking with music was expected to be more rewarding than with a metronome.^25^ Moreover, patients with PR to cueing did not report being in general more engaged in musical tasks or emotionally driven than the others. An alternative explanation, which deserves further inquiry, is that auditory stimuli altogether are more arousing while walking as compared with no stimulation.^26^

The immediate response to cueing was linked to some aspects of patients’ musicality, such as self-assessed perception and musical training. The activities engaging the neural circuitries for beat perception such as learning to play an instrument or singing might thus be put to use in rehabilitation. Whether previous musical training influences the specific neuronal pathways underpinning the beneficial effects of cueing is an open question. Rhythmic cues provide a regular temporal scaffolding supporting motor coordination (e.g., by directing patients’ attention towards the onsets of individual steps), thus probably compensating for patients’ impaired internal timing. The underlying mechanism would be underpinned by compensatory cortico-subcortical networks such as cerebello–thalamo–cortical circuitries,^3,5,27^ typically affected only later in the disease, or by the residual activity of cortico-striatal networks.^3,5^ Patients having received some form of musical training may be better equipped to benefit from RAC than non-musicians. In sum, musical experience, as observed in other neurodegenerative disorders or in stroke,^2^ may play a neuroprotective role in PD.

Cognitive and psychopathological evaluation in these non-demented patients did not reveal differences between patients with PR and NPR to cueing. The only exception was the Wisconsin Card Sorting Test, in which patients with PR performed worst than patients with NPR to cueing. Patients who positively responded to cueing showed lower cognitive flexibility as compared to the other patients. This reduced flexibility could facilitate the maintenance of a constant gait pattern while walking with a cue, and thereby be one of the determinants of patients’ PR to the stimulation. This possibility is supported by the finding that lower cognitive flexibility is associated with better synchronization of the steps to the beat.

This study has a few limitations. The sample size, albeit this is quite large when considering the entire group of patients, is small when studying subgroups of patients with positive and non-positive response to cueing. Moreover, the exclusion of patients with high risk of falling (i.e., by selecting those patients who might use self-rehabilitation programs with music at home), and patients with freezing of gait reduces the generalization power of our findings. Nevertheless, note that patients who are more impaired (e.g., with lower gait speed at baseline) are likely to benefit more from cueing^4^ than less impaired patients. Finally, the exclusion of patients with dementia might have reduced the cognitive differences observed in the different groups. Future studies should be devoted to testing the role of the predictors we identified in more severe patients, in terms of their motor and cognitive impairments. It is expected that the factors we highlighted as potential predictors of positive reponse to cueing may play even a more important role in more severe patients.

In sum, these findings show that patients with some degree of musical training and who display good beat perception and thereby spontaneously align their steps to the beat are ideal candidates for RAC as a rehabilitation strategy, as also suggested by a previous training study.^4^

In contrast, patients who perform poorly in rhythmic tasks are at risk of experiencing deleterious effects of cueing on gait, with a reduction of gait speed and stride length, thus potentially increasing the risk of falling and dependency.

## Methods

### Participants

Thirty-nine non-demented patients (24 males, 62 ± 10 years old) with PD and gait disorders were recruited at the Neurology Department of Beau Soleil Clinic and University Hospital of Montpellier (France). PD diagnosis was established according to the Queen Square Brain Bank criteria. Gait abnormalities were defined when the patients were in ON-state as lower limb akinesia inducing asymmetry of steps, reduction of step length, or a reduction of speed. Patients with severe gait initiation failure or postural instability were excluded because of increased risk of falls. Moreover, patients with freezing of gait were excluded because of the different pathophysiology and the risk of falls associated with this gait disorder. The control group was formed by 39 gender-matched, age-matched, and education level-matched healthy controls recruited via the database of the Clinical Investigation Centre of the Montpellier University Hospital. Patients and controls with hearing impairment were excluded. All participants provided written informed consent prior to the experiment. The study was approved by the National Ethics Committee (CPP Sud Méditérannée III, Nîmes, France, ID-RCB: 2014-A00021-46).

### Clinical and neuropsychological tests

Data concerning demographic characteristics, medical history, course of PD, and treatment were collected during a preliminary interview. Motor severity of the disease was evaluated on the Hoehn and Yahr scale^28^ and using the revised Movement Disorder Society-Unified Parkinson’s Disease Rating Scale part III (MDS-UPDRS-III)^29^ when in “ON” state. The levodopa equivalent daily dose was calculated.^30^ Self-evaluation of the risk of falls was provided by the patients using the Falls Self-Efficacy Scale Score.^31^ Non-motor and motor experience of daily living was evaluated using MDS-UPDRS parts I and II, respectively, and motor complications using part IV.^29^

Global cognitive functioning was tested with the Montreal Cognitive Assessment.^32^ Attention and executive functions were assessed with the Digit Span subtest from Wechsler Adult Intelligence Scale version III (WAIS-III; scaled score),^33^ the Trail Making Test part B,^34^ and a modified version of the Stroop Colour Word Test (part III - part I, time in seconds; part III - part I number of errors.^35^ To take into account difficulties in lexical access, a naming score was established as [(naming time – reading time)/reading time] × 100. To examine executive processes, we calculated an interference score as [(interference time – naming time)/naming time] × 100. To better dissociate the effect of akinesia from the temporal increase due to other cognitive processes for the Trail Making test a ratio was calculated corresponding to trail making test B/trail making test A.^36^ Mental flexibility was assessed using the Wisconsin Card Sorting Test.^37^ Depressive symptoms were tested with the Beck Depression Inventory,^38^ and apathy using the Lille Apathy Rating Scale.^39^

### Cueing and gait recording

Auditory cues were a metronome and musical excerpts from four military marches.^40^ The rate of auditory cues was set to 10% faster than each participant’s preferred cadence, measured at pre-test.

Gait spatio-temporal parameters were recorded via sensors (inertial measurement units including 3D accelerometers and gyroscopes, MobilityLab®, APDM Inc., Portland) strapped over the feet and anterior side of left and right tibia, and sternum. Gait variability and the phase coordination index (PCI) were computed.^40,41^

### Testing of rhythm abilities and musicality

Participants’ rhythm abilities were measured with the Battery for the Assessment of Auditory Sensorimotor and Timing Abilities (BAASTA).^42^ Rhythm perception was tested with the Beat Alignment Test.^43^ Rhythm production was measured with unpaced and paced finger tapping tasks.

A “synchronization score” in gait and tapping, indicating how well participants aligned their movements to the beat was calculated, varying from 0 (no synchronization) to 1 (maximal synchronization).^40,44^

Subjects’ musicality was assessed with the Gold-MSI (Goldsmiths Musical Sophistication Index).^1^ This is a 39-item self-report inventory for self-reported musical skills, divided into five subscales that allow assessing active engagement, perceptual abilities, musical training, singing abilities, and emotions.

#### Statistical analyses

To ensure adequate power, the chosen sample size of patients was comparable or larger than in previous studies showing an effect of cueing on gait (e.g., 11). Patients with PD were compared to controls for demographic and neurological variables using *t*-tests. As no effect of the type of rhythmic stimulus was observed on gait,^17^ data were pooled before running subsequent analyses.

Spatio-temporal gait parameters were entered in 2 × 2 mixed-design ANOVAs with Group (patients vs. controls) as between-subject factor and Condition (baseline vs. cueing) as within-subject factor. As no significant interactions were found in the ANOVAs only main effects of Group (patients vs. controls) and Condition (cueing vs. baseline) are reported. Normality of distributions was assessed with Shapiro-Wilk tests, and heteroscedasticity with Bartlett tests of homogeneity of variances. Tests revealed that in most of the cases the two assumptions were met. Participants with PR and NPR to cueing were compared via 2 × 2 × 2 ANOVAs using Group (patients vs. controls) and Response (PR vs. NPR) as between-subject factors, and Condition (baseline vs. cueing) as the within-subject factor. Whenever the triple interaction was significant (*p* < 0.05), the interaction was decomposed by running separate 2 × 2 ANOVAs for patients and controls. For the assessment of rhythm perception and production, synchronization, and musicality, patients’ individual performances were transformed into *z*-scores based on the mean and standard deviation of controls. Transformation into *z*-scores was performed separately for individuals with PR and NPR to cueing. Finally, in order to compare patients with NR to cueing (*n* = 6) to all other patients, given the small sample size, non-parametric tests were used (Wilcoxon rank sum tests).

### Data availability and sharing statement

Our data are available and we can share them if asked.
